# Genome-Wide Analysis of Long Non-Coding RNA Profiles in Canine Oral Melanomas

**DOI:** 10.3390/genes10060477

**Published:** 2019-06-23

**Authors:** Christophe Hitte, Céline Le Béguec, Edouard Cadieu, Valentin Wucher, Aline Primot, Anaïs Prouteau, Nadine Botherel, Benoît Hédan, Kerstin Lindblad-Toh, Catherine André, Thomas Derrien

**Affiliations:** 1 University of Rennes, CNRS, IGDR—UMR 6290, F-35000 Rennes, France; celine.lebeguec@gmail.com (C.L.B.); edouard.cadieu@univ-rennes1.fr (E.C.); aline.primot@free.fr (A.P.); anais.prouteau@univ-rennes1.fr (A.P.); nadine.botherel@univ-rennes1.fr (N.B.); benoit.hedan@univ-rennes1.fr (B.H.); catherine.andre@univ-rennes1.fr (C.A.); 2 Centre for Genomic Regulation (CRG), The Barcelona Institute of Science and Technology, Dr. Aiguader 88, 08003 Barcelona, Spain; v.wucher@gmail.com; 3 Broad Institute of MIT and Harvard, Cambridge, MA 02142, USA; kersli@broadinstitute.org; 4 Science for Life Laboratory, Department of Medical Biochemistry and Microbiology, Uppsala University, Box 582, SE-751 24 Uppsala, Sweden

**Keywords:** mucosal melanoma, dogs, transcriptome sequencing, long non-coding RNAs (lncRNAs)

## Abstract

Mucosal melanomas (MM) are rare aggressive cancers in humans, and one of the most common forms of oral cancers in dogs. Similar biological and histological features are shared between MM in both species, making dogs a powerful model for comparative oncology studies of melanomas. Although exome sequencing recently identified recurrent coding mutations in canine MM, little is known about changes in non-coding gene expression, and more particularly, in canine long non-coding RNAs (lncRNAs), which are commonly dysregulated in human cancers. Here, we sampled a large cohort (*n* = 52) of canine normal/tumor oral MM from three predisposed breeds (poodles, Labrador retrievers, and golden retrievers), and used deep transcriptome sequencing to identify more than 400 differentially expressed (DE) lncRNAs. We further prioritized candidate lncRNAs by comparative genomic analysis to pinpoint 26 dog–human conserved DE lncRNAs, including *SOX21-AS*, *ZEB2-AS*, and *CASC15* lncRNAs. Using unsupervised co-expression network analysis with coding genes, we inferred the potential functions of the DE lncRNAs, suggesting associations with cancer-related genes, cell cycle, and carbohydrate metabolism Gene Ontology (GO) terms. Finally, we exploited our multi-breed design to identify DE lncRNAs within breeds. This study provides a unique transcriptomic resource for studying oral melanoma in dogs, and highlights lncRNAs that may potentially be diagnostic or therapeutic targets for human and veterinary medicine.

## 1. Introduction

Mucosal melanomas (MM) are the most frequent form of melanomas in dogs, and they display more aggressive behavior in comparison to cutaneous melanomas. Dogs are spontaneously affected, with specific breeds developing MM with clinical features that are similar to human melanomas [1]. Dog breeds with high melanoma risk have been proposed as relevant natural models for the comparative oncology of melanomas, especially for deciphering their non-UV-dependent pathways, and for developing clinical trials that are based on homologous melanoma subtypes [1,2].

Recently, genomic studies have been conducted, to identify driver genomic alterations that are involved in canine MM [3,4], using exome sequencing to focus on the genetic landscape of somatic mutations in protein-coding genes (messenger RNAs; mRNAs). A consequence of cumulative genetic and epigenetic alterations in coding and non-coding genes is reflected by the study of gene expression, which has not yet been investigated in detail in canine model cancers. Despite the recent identification of thousands of canine long non-coding RNAs (lncRNA) [5,6], little is known about their impact in dog cancers, although they constitute an extensive component of dog genomes [7,8,9]. In humans, lncRNA expression is recurrently altered in many types of cancers [10,11,12], including melanomas [13]. From the first annotation of the melanoma-associated lncRNA *SPRY4-IT1* [14] to the recent identification of recurrent amplifications of *SAMMSON*, a dozen of lncRNAs have been functionally validated in cutaneous melanomas [15]. Because lncRNAs are expressed in a tissue-specific manner in both humans [8,16] and dogs [6], they represent a vast and still unexplored repertoire of potential targets and/or biomarkers for comparative oncology approaches.

Here, we analyzed a large cohort of canine MM transcriptomes from three breeds, sampled from the oral cavity (*n* = 52). We quantified differential gene expression by controlling for cell heterogeneity, using a signature-based method, and we assessed transcriptional networks by using co-expression analysis. We showed that lncRNA expression profiles discriminate between normal and tumor samples, and identified a significant amount of deregulation for 400 lncRNAs. Gene-set enrichment analyses were performed using co-expression networks of lncRNA:mRNA, to acquire associated GO enrichments for all-breed and breed-specific DE lncRNAs. Furthermore, we conducted dog-human orthologous relationship analyses to identify conserved lncRNAs, with potential interest in human melanomas. Altogether, this study provides an in-depth characterization of lncRNAs that are dysregulated in canine oral melanomas, and prioritizes potential biomarker lncRNAs by investigating their conservation and co-expression networks. Our findings provide a novel transcriptomic resource with detailed sample characterization for the comparative oncology of melanomas in dogs and humans.

## 2. Materials and Methods

### 2.1. Canine RNA Samples: Extraction and Sequencing

In total, 39 dogs from three breeds (GRET: golden retrievers, LABR: Labrador retrievers, and PODL: poodles) were sampled with either tumor-only (*n* = 26) or matched tumor/normal samples (*n* = 13 × 2) (totaling 52 samples) from the two biobanks, “Cani-DNA_BRC”, which is part of the CRB-Anim infrastructure, and the Canine Comparative Oncology and Genomics Consortium (CCOGC). Samples were collected in the course of the health management of the dogs, by DVM veterinarians, with the owner’s consent, and the diagnosis was performed through histopathological analyses (CNRS ethical board, France (35-238-13)). Material was collected at surgery, then stored in RNAlater, and the diagnosis of mucosal melanoma was evaluated by specialized veterinarians after histological examinations of the samples.

For all of the 52 samples, RNAs were extracted by using RNA II NucleoSpin Kits according to the manufacturer’s instructions (Macherey-Nagel, Hoerdt, France) then polyadenylated RNAs (polyA+) were selected and sequenced at the BROAD sequencing platform, in a paired-end and stranded fashion, using HiSeq-2000 Illumina technology (BROAD Genomics Platform, Cambridge, MA, USA), at a mean depth of 107.4 million reads per sample. The RNA-Seq data is available in European Nucleotide Archive.

We used the “canFam3.1-plus” annotation [5,6] containing 10,444 lncRNAs and 21,810 protein-coding genes as the reference annotation, and the canFam3.1 assembly version as the genome reference [16]. Based on the protocol described in Djebali et al. [17], FASTQ reads were aligned, both on the transcriptome and on the genome, using the STAR program (v2.5.0a) [18]. Finally, gene and isoform expression levels were estimated in both normalized (TPM: transcripts per million) and un-normalized (raw count as required by DE tools) with the RSEM program (v1.2.25) [19] for each sample individually, and then merged in order to obtain a matrix expression file, with genes in rows and samples in columns.

### 2.2. Analysis by DESeq2 using a Multi-Factor Design including Cell-Type Heterogeneity

The matrix of reads counts, including lncRNAs and mRNA genes, was used by DESeq2 (v1.22.2) [20] to compute differentially expressed genes. Given the cellular heterogeneity between healthy and tumor samples, we used the xCell program (v1.12) [21] to compute cell-type enrichment from our gene expression data, based on the reference signature set of 64 immune and stroma human cell types (Supplementary Materials Figure S1). The cell-type enrichment scores for keratinocytes, melanocytes, and skeletal muscle cells were then included in the DESeq2 design, in order to specifically control for DE genes involved in the tumor condition, and not in the differentiation between cell types (e.g., keratinocytes versus melanocytes). To control for other covariates, we included breed and sex information in our design, resulting in the following DESeq2 formula: design = ~sex + cell_types + breed + condition, with the condition here being the status of the sample (with normal tissue being a control for cell type heterogeneity versus tumoral tissue). To take into account low gene counts (which are especially the case for lncRNAs), we used the recently developed lfcShrink method with the type = apeglm option [22] to better estimate log-fold changes for poorly expressed genes. To test whether the log-fold change linked to oral melanoma was different between breeds, we added an interaction to the design, such as breed:condition.

### 2.3. Identification of Human Orthologous lncRNAs

For each canine lncRNA gene belonging to the “canFam3.1-plus” annotation, we projected all of its exons onto the canine genome, resulting in one representative “meta-transcript” sequence per gene. These sequences were then mapped onto the human genome assembly version GRCh38, using minimap2 [23] with the following parameters -ax splice -t16, and only primary alignments being retained in the case of multiple mappings. Based on the CIGAR field, sequence identity was defined as the number of matching bases over the number of alignment columns. Finally, human orthologous coordinates were compared with the GENCODE (v29) annotation of the lncRNA exons [8,24] using the bedtools [25] intersect program (after BAM to BED12 file format conversion) with the following parameters: -s -split, in order to assign orthologous relationships.

### 2.4. Weighted Gene Coexpression Network Analysis

A weighted gene coexpression network analysis (WGCNA) was carried out on the 52 RNA-Seq reads, using the R package WGCNA 1.66 [26]. The program utilizes a similarity measure to summarize the relationship between all pairs of genes, using expression data normalized as TPM to create a correlation matrix. We used the signed WGCNA coexpression measure. To identify coexpression modules, we used the ‘soft-thresholding procedure’. WGCNA utilizes a similarity measure to summarize the relationship between all pairs of gene expression data across the data set, to create a correlation matrix. Co-expression modules are defined as branches of a cluster tree, using a dynamic branch-cutting approach. Therefore, co-expression modules are clusters of co-expressed genes identified by hierarchical cluster analysis.

Constructing a weighted gene network entails the use of a soft-threshold score that assigns a connection weight to each gene pair. The co-expression similarity is raised against the soft thresholding power, in order to calculate adjacency. For soft thresholding, we used the two adjacency functions that convert the co-expression measure to a connection weight. First, the scale-free fit index is a function of the soft-thresholding power. Second, the mean connectivity is a function of the soft-thresholding power. We set the soft-threshold to 7, to avoid the selection of an arbitrary cut-off. The weighted separation of co-expression was achieved by the transformation of the correlation matrix in an adjacency matrix, using default values. Gene profiles that had a low expression and/or did not vary sufficiently across each of the data sets were eliminated. A total of 3,830 genes met these criteria. We performed principal component analysis, and used the first principal component (module eigengene; ME) to summarize the standardized module expression data.

To assess the potential associations between coexpressed gene modules and the melanoma condition, a single-column vector of clinical data for each breed and for all breeds considered together was defined and utilized. An association analysis was performed by using the module-trait WGCNA method to perform correlation analysis of the ME with clinical traits. Correlations and the corresponding *p*-values allowed for an inspection of the most significant associations.

Intramodular analysis was performed to identify genes with high gene significance and module membership measures, as recommended by WCGNA procedures. Genes with high significance (>0.5) for each variable, as well as high module membership (>0.5) in interesting modules were extracted.

### 2.5. Gene Set Enrichment Analysis

We conducted Gene Set Enrichment Analysis using the GSEA webserver [27], to construct meaningful annotation from the GO of genes (mRNAs), defined a priori by the WGCNA modules. The ontology that was used covered the domain of biological processes (BP).

## 3. Results

### 3.1. Whole-Transcriptome Sequencing of Oral Melanomas

We sampled 39 oral melanomas from three breeds (16 golden retrievers, 13 Labrador retrievers and 10 poodles) that were classified with respect to their oral melanoma locations, which included the tongue for 26% of the annotated cases, followed by the maxilla (18%) (Supplementary Materials Table S1).

Combining healthy and tumor samples, 5.7 billion sequencing reads were generated, with an average of 107 million reads per sample (Supplementary Materials Table S2). After quality control and trimming of the adapters, between 89% and 96% of the reads could be mapped onto both the canine genome assembly (canFam3.1) and the “canFam3.1-plus” annotation [5,6], using the state-of-the-art bioinformatic protocol described in Djebali et al. [17]. Amongst the lncRNA genes, we focused on long intergenic ncRNAs (lincRNA; *n* = 5651) and antisense lncRNAs (antisense; *n* = 4793), thus removing sense intronic lncRNAs which may correspond to the misannotation of coding alternative isoforms, and observed that 59.0% and 58.5% respectively could be considered as being expressed, using a soft filter of 10 reads in total per gene. In comparison, 87.6% of the total number of protein coding genes (*n* = 21,810) were retained, using the same threshold.

### 3.2. Analysis of Differentially Expressed Genes (DEG) in Mucosal Melanomas

**Figure 1:**
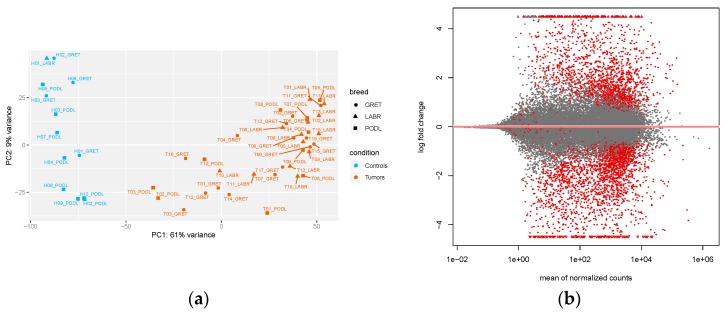
Expression analysis of the 52 oral melanoma samples. (**a**) Principal component analysis (PCA) of the 52 samples, based on gene-normalized counts, with control and tumor samples in blue and orange, respectively; (**b**) M-A plot representing log2-fold gene changes between tumors and controls over the mean of the normalized counts, with red points corresponding to significantly DE genes, with an adjusted *p*-value < 0.05, and without a log-fold change threshold; genes falling outside of the window are plotted as open triangles.

We first performed quality control of the samples by using a PCA with all gene counts, as normalized by the DESeq2 program (size factors normalization) (Figure 1a). This revealed a clear distinction of the samples, with the first principal component distinguishing the control from the tumor samples in the three breeds. A similar distribution was observed when taking into account only the lncRNA-normalized counts, although the percentage of the explained variance was slightly lower (Supplementary Materials Figure S2). We next used DESeq2 to identify differentially expressed genes (both lncRNAs and mRNAs) by controlling for specific covariates: breed, sex, and cell-type heterogeneity between the samples (see Methods). For the latter, expression data was incorporated into the xCell program [21], and samples were then clustered according to their enrichment within the 64 cell-type signatures used by the program (Supplementary Materials Figure S1). Control samples were found to be enriched in keratinocyte-like and skeletal muscle cells, while tumor samples tended to be enriched in melanocyte cells. Using this multi-factor experimental design, we identified 417 differentially expressed lncRNAs between tumor and control samples, using an absolute log2 fold-change (|lFC|) > 1.5, and an adjusted *p*-values (*p*_adj_) < 0.05 (see methods) (Figure 1b). From a cross-check of the DE analysis, we found that the *MDM2* proto-oncogene, shown to be recurrently gained in human non-cutaneous melanomas [28], was almost four times more highly expressed in canine oral melanoma tumors than in controls (lFC = 1.96; *p*_adj_ = 0.02). Similarly, we observed a significantly lower expression of the *BUB1* gene in our cohort of canine melanomas (lFC = −1.06; *p*_adj_ = 0.02), in accordance with recent findings showing recurrent deletions of *BUB1* in mucosal melanomas using a cross-species strategy, including human, horse, and dog samples [3]. Among the 417 DE lncRNAs, 272 lncRNAs were down-regulated and 145 were up-regulated.

Although most canine lncRNAs have not yet been functionally characterized, one notable exception was given by the lncRNA *ZEB2-AS*, transcribed in an antisense orientation to the *ZEB2* mRNA, which was almost 14 times more highly expressed in tumors compared to normal tissues (lFC = 3.79, *p*_adj_ = 2.7 × 10^−8^). Interestingly, this lncRNA has been shown to be involved in the regulation of *ZEB2* mRNA during the epithelial–mesenchymal transition (EMT) in human cell lines [29].

### 3.3. Comparative Genomics of Canine Differentially Expressed lncRNAs

**Figure 2:**
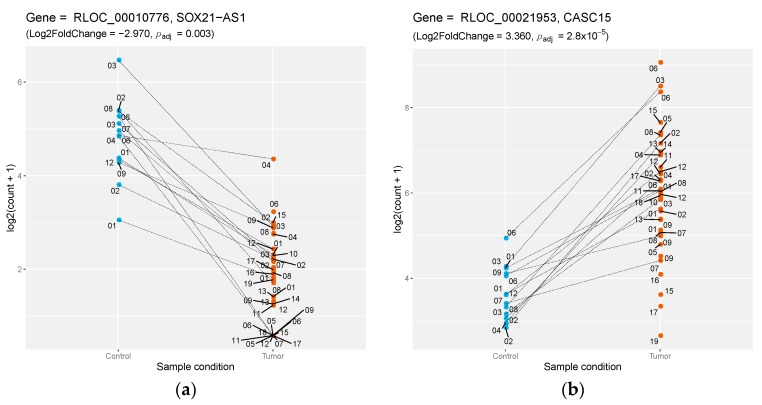
Differential expression of dog–human-conserved lncRNAs. (**a**) Down-regulation of the *SOX21-AS1* lncRNA between control samples (blue) versus tumor samples (orange), with the log2 of normalized counts on the *y*-axis; lines connect matched samples from the same individuals. (**b**) Same representation for the up-regulation of the lncRNA *CASC15*.

Previous comparative genomic analysis [6] allowed us to identify a set of orthologous lncRNAs between human and dog, using a synteny-based approach. Here, we sought to annotate novel orthologous lncRNAs between dog and human by directly mapping DE lncRNAs sequences onto the human genome, using the minimap2 program [23] (see Methods). With the human genome assembly version GRCh38 defined as the target sequence, we aligned 33% of canine DE lncRNAs (*n* = 140) with a minimum identity of 70%. Amongst those, 26 matched to an already GENCODE-annotated [24] non-coding gene (Table 1). Most notably, we showed that several cancer-associated annotated lncRNAs in human are differentially expressed in canine mucosal melanomas. This included the down-regulation of *SOX21-AS1* (lFC = −2.97, *p*_adj_ = 0.003) (Figure 2a), already shown to be silenced in oral cancers [30], and the overexpression of the *CASC15* gene (Cancer Susceptibility Candidate 15) (lFC = 3.3, *p*_adj_ = 2.8 × 10^−5^) (Figure 2b), whose RNA level has also been linked to cutaneous melanoma and phenotype switching in humans [31]. This analysis also shed light on 114 canine DE lncRNAs, which aligned to the human genome (identity > 70%) but without any known annotated transcripts by GENCODE, potentially highlighting novel human lncRNAs (Supplementary Materials Table S3).

### 3.4. Inferring Functions of Differentially Expressed lncRNAs

We conducted an unsupervised expression analysis of lncRNAs, utilizing a WGCNA [26] based on the 52 RNA-Seq samples. The advantage of WGCNA is that it transforms gene expression data into co-expression modules, providing insights into signaling networks that may be responsible for the development and progression of oral melanomas. We included protein-coding genes (*n* = 21,810) to identify coexpressed modules that reveal relationships between lncRNAs and mRNAs, suggest common biological roles, and inform potential roles for lncRNAs.

#### 3.4.1. Correlating Transcriptional Networks and Traits using Co-Expression Analysis

**Figure 3:**
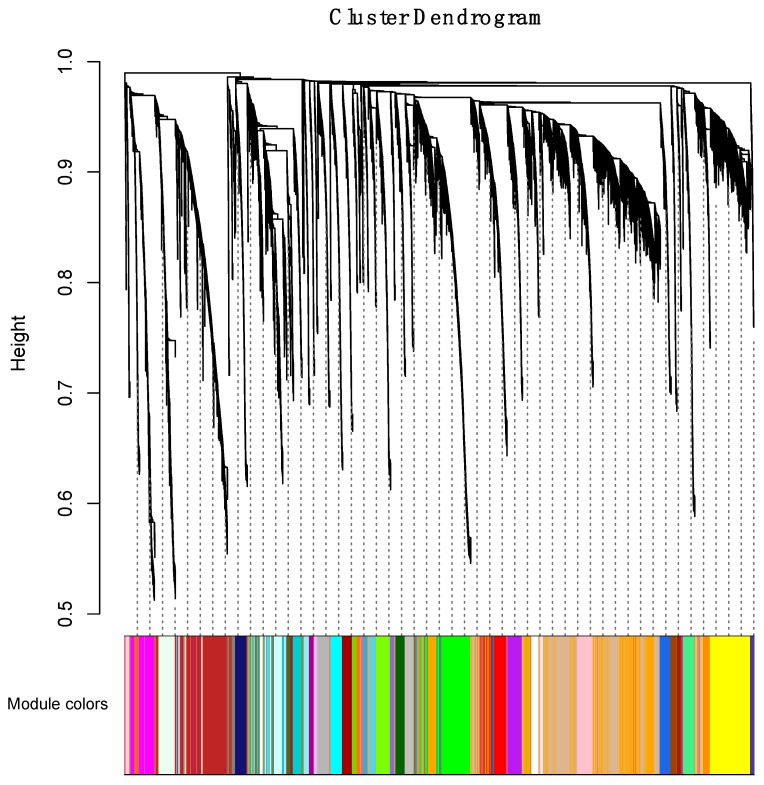
Clustering dendrogram. A total of 59 coexpression modules were constructed with assigned module colors at the bottom. The number of lncRNAs in the 59 modules is listed in Supplementary Materials Table S4.

In the initial phase of the WGCNA, we identified 59 coexpression modules in an unsupervised manner. Hierarchical clustering analysis was performed, and a dendrogram was used to represent coexpression modules, as shown by color assignments (Figure 3). The coexpression modules included 121 lncRNAs on average (a range of 10 to 627).

**Figure 4:**
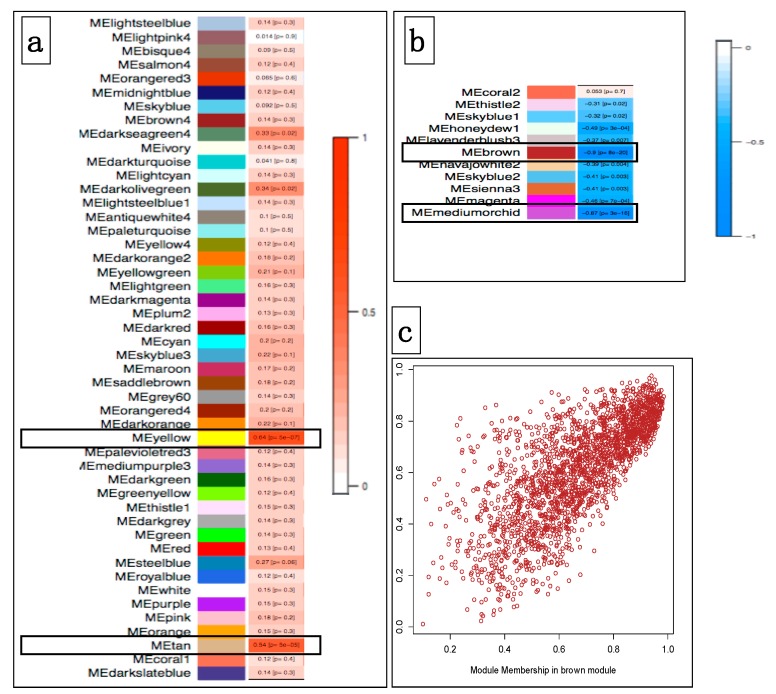
Module–trait associations. (**a**) Each row corresponds to a ME (module eigengene), and the column to the mucosal melanoma trait. Each cell contains the corresponding correlation and *p*-value with melanoma. Each correlation is color-coded according to the strength of the correlation, with a red gradient for positive correlations (red bar in 4.a). Modules Yellow and Tan are positively correlated (*p* < 5 × 10^−5^). (**b**) Modules with negative correlations according to the strength of the correlation; (blue bar in 4.b). Module Brown and Medium-orchid are the most significantly negatively correlated (*p* < 1 × 10^−16^). (**c**) Scatterplot of gene significance for mucosal melanoma status vs module membership for the Brown module. It shows a highly significant correlation between gene significance and Module membership in this module.

We further performed the identification of coexpression modules that are associated with oral mucosal melanoma from all samples, through the calculation of Pearson’s correlation coefficient (PCC). We further carried out intramodular analysis to identify genes with the highest significance association with MM, as well as a quantitative measure of membership in the module given by the correlation of the eigengene module with the gene expression profile. We identified four modules (hereafter termed brown, medium-orchid, yellow, and tan) that were significantly associated with the melanoma status, with two modules being positively associated (PCC = +0.64, *p* = 6 × 10^−7^ ME yellow; PCC= +0.54, *p* = 5 × 10^−5^ for ME Tan), while the two other modules showed significant but opposite PCC associations with melanoma (ME brown, PCC = −0.90, *p* = 8 × 10^−20^; ME medium-orchid, PCC = −0.87, *p* = 3 × 10^−16^) (Figure 4).

We considered only lncRNAs identified by the DE analysis in the coexpression analysis (*n* = 417) to overcome the heterogeneity bias between tumor and control cell types. A total of 215 DE lncRNAs (51.5%) also belonged to coexpressed modules with significant PCCs with melanoma status, such as the dog–human-conserved lncRNA *SOX21-AS1* which was found to be down-regulated in dog MM. In light of their correlations with cancer, dysregulated lncRNAs were classified into two categories; 30 belonged to modules with significant positive correlations, and 185 were in modules that yielded significant although opposite PCC.

#### 3.4.2. Using Transcriptional Networks for Inferring lncRNA Functions

**Figure 5:**
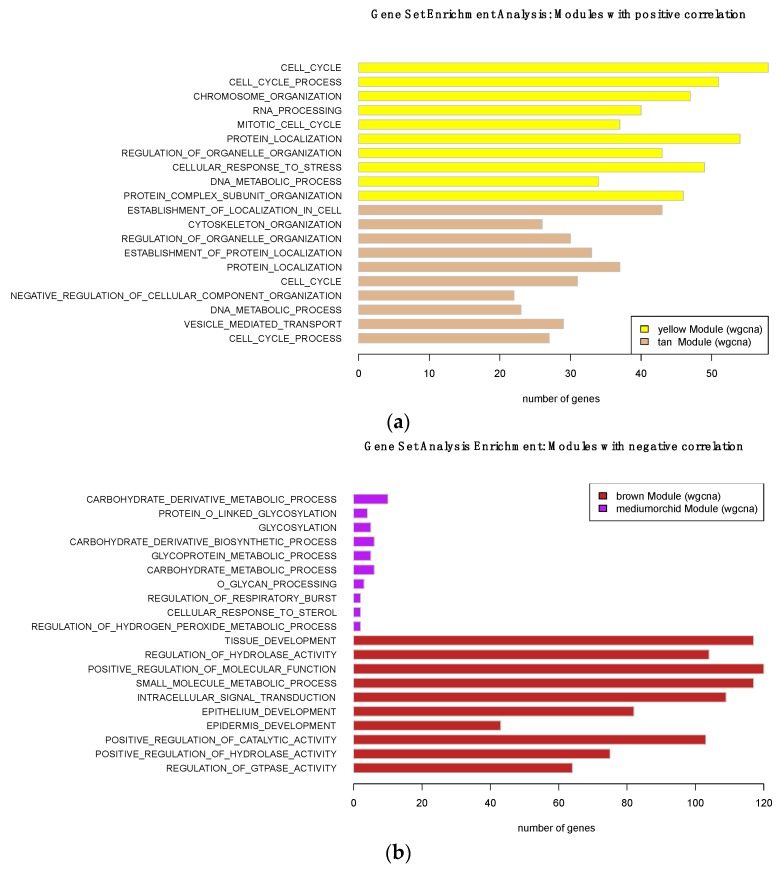
GO terms (Biological Process) enriched for (**a**) positively correlated and (**b**) negatively correlated modules with oral melanoma: the top ten enriched GO items are represented.

We used the lncRNA:mRNA correlated transcriptional networks constructed by WGCNA to infer the main functions of the lncRNAs, using the ‘guilt-by-association’ principle [32]. The functional implication of coexpressed mRNAs within the four modules (brown, medium-orchid, yellow, and tan) that were significantly associated with MM was evaluated via gene set enrichment analysis, using the GSEA tool [27]. Our data showed that both positive and negative modules were significantly associated with specific but distinct GO biology process terms. As shown in Figure 5, genes involved in the positively associated module were enriched for GO terms involved in “cell cycle”, “cell cycle process” or “mitotic cell cycle” for the yellow module, and “chromosome organization”, “cellular response to stress”, and “DNA metabolic process” for the tan module. These GO terms are connected with cancer, and implicated the replication and segregation of genetic material, and progression through the phases of the mitotic cell cycle.

Conversely, genes of the negatively correlated modules were mainly enriched in “tissue development”, “epithelium development”, and “epidermis development” for the brown module, and mostly in “carbohydrate metabolic process” for the medium-orchid module. These categories reflect processes whose specific outcomes are the progression of a tissue over time, from its formation to its mature structure, and many pathways involving carbohydrate derivatives.

#### 3.4.3. Breed-Specific lncRNAs Associated with Oral Melanoma

The design of our study, which included three distinct breeds predisposed to MM, made it possible to integrate both the coexpression module analysis and the differentially expressed lncRNAs, for each separate breed. Given the low number of control samples for Labrador retrievers, we focused our analysis on the pairwise comparisons between golden retrievers and poodles. Using WGCNA, the analysis of the poodle breed produced a significant correlation for eight modules (six with PCC > 0.8, *p* < 2 × 10^−12^ and two PCC < −0.8, *p* < 2 × 10^−15^) (Supplementary Materials Figure S3). From these modules, the gene set enrichment analysis showed that the GO terms (biological process) that were most significantly enriched were “regulation of gene expression”, “chromatin organization”, and “chromatin modification” (orange module, Supplementary Materials Figure S4). Complementary to this analysis, we refined the DESeq2 experimental design, which previously computed the global melanoma effect while controlling for differences due to the breeds, to search for DE lncRNAs only in poodles, and not in golden retrievers (see Methods). Our analysis identified a panel of 11 lncRNAs that were significantly DE only in poodles (|lFC| > 1.5 and *p*_adj_ < 0.05), and which belong to WGCNA modules associated to poodles (Supplementary Materials Table S5). For instance, we observed that the most significant DE lncRNA (*RLOC_00005829*) is down-regulated in poodles (lFC = −5.99, *p*_adj_ = 8.1 × 10^−7^), while its expression is not significantly altered in golden retrievers (*p*_adj_ = 0.61) (Supplementary Materials Figure S5). Concordantly, this lncRNA was not considered as being DE (*p*_adj_ = 0.54) in the first design when the tumor effect was controlled for differences due to breeds. Finally, we mapped the *RLOC_00005829* sequence on the human genome, and showed that it clearly aligned to the *COLCA1* gene (identity = 61.1%), a GENCODE-annotated antisense lncRNA [24] that was already associated with human colorectal cancer by GWAS [33].

For golden retrievers, the coexpression analysis produced significant correlations for four modules (PCC < −0.8, *p* < 3 × 10^−20^). Similarly, the DE analysis identified a panel of seven lncRNAs only found to be differentially expressed in golden retriever samples and not in poodles, but these were not identified with WGCNA (Supplementary Materials Table S5).

## 4. Discussion and Conclusions

Long non-coding RNAs (lncRNAs) are key regulators in many biological processes and they are often dysregulated in cancers [34], including cutaneous melanoma [13]. We investigated lncRNAs of the canine model as being potential cancer markers for mucosal melanomas in humans. Our findings show the existence of a genetic basis and expression variation involving long non-coding RNAs in oral mucosal melanomas in dogs from three breeds (golden retrievers, Labrador retrievers, and poodles), with an increased risk of developing oral mucosal melanomas.

In this study, bioinformatic analyses identified more than 400 dysregulated lncRNAs that discriminated canine oral melanoma tumors from control samples. We further pinpointed one down-regulated (*SOX21-AS*) and two up-regulated (*CASC15* and *ZEB2-AS*) DE lncRNAs (inferred as “onco-lncRNAs”) [35,36] in canine oral melanoma, that were significantly conserved with humans, and already associated with human cancers. These results provide a novel resource for candidate biomarkers, for which further in vitro and in vivo experimental validations will be required.

Although we used bulk RNA-Seq to analyze dysregulated lncRNAs in canine oral melanomas, we adopted an enrichment-based computational analysis to control for covariates such as cell-type heterogeneity between samples. Importantly, the xCell program, which was used to compute these enrichments, includes melanoma-related cell-types from the Tirosh et al. single-cell RNA-Seq study [37], such as malignant, immune, and endothelial cells. In melanomas, the distinct subtypes that most likely harbor multiple cell types and high genetic heterogeneity are thought to play a role in the development and progression of tumors. Future directions to explore the distinct genotypic and phenotypic states of the tumors will involve directly performing single-cell RNA sequencing on oral mucosal melanomas. Furthermore, the expression of lncRNAs is highly tissue- and cancer-specific [8,34] and this is particularly relevant for studying cells of the same tumor and/or tissue that exhibit transcriptional heterogeneity [38]. In our study, we also observed that the tissue specificity, measured from canine normal tissues [6], was significantly higher for DE lncRNAs than for DE mRNAs (*p*-value= 2.8 × 10^−9^, Wilcoxon’s rank-sum test), reinforcing the attractiveness of lncRNAs as potential biomarkers of oral melanomas.

We also report a weighted gene coexpression network analysis (WGCNA) that constructed 59 modules by an unsupervised analysis of gene expression profiles. The WGCNA method was further used to detect the relationship between the lncRNA expression profiles and the melanoma status. WGCNA has many advantages over other clustering methods, since the analysis uses a ‘soft-thresholding procedure’ to avoid the selection of an arbitrary cut-off. It also focuses on the association between coexpression modules and clinical features, and the results have robust reliability and biological significance. Genes in the same module are considered to be related with each other by their functions. We identified four coexpression modules that are related to oral melanoma for all breeds studied, and specific DE lncRNAs for the poodle and golden retrievers breeds. Thus, this study led to the identification of biologically relevant modules and hub lncRNAs that could serve as biomarkers for the detection of mucosal melanomas [39].

To give biological meaning to identify lncRNAs, we conducted a gene set enrichment analysis. These analyses showed clear differences in enriched GO (BP) terms between the different modules, which were largely associated with different functions. As a result, modules containing up-regulated genes were found to be mainly enriched in cancer-associated pathways, implicating the replication and segregation of genetic material, and its progression through the mitotic cell cycle phases. The dysregulated lncRNAs of these modules could possibly have a role in cell cycle or cell proliferation. Modules with down-regulated genes were largely involved in carbohydrate metabolic processes. Carbohydrates and glucose can have important effects on the proliferation of tumor cells. It has been reported that most malignant cells are dependent on the availability of glucose in the blood for their energy, and that they are not able to metabolize it, especially in case of mitochondrial dysfunction [40].

Gene expression profiling is actively investigated as a clinical biomarker and diagnostic tools to detect multiple cancer types and distinct stages. However it is challenging to take into account the variability of gene expression, and thus the underlying functions of genes in populations of different ethnic origins [41]. Here, we used the unique features of the dog model, and its diversity and breed structure, to study the expression variations of lncRNAs that are associated with mucosal melanomas between breeds. We have identified lncRNAs that are differentially expressed only in melanomas sampled in poodles, such as the antisense lncRNA *COLCA1*. Therefore, the variation in lncRNA expression identified in dog breeds may help to better characterize the observed disparities and heterogeneity of mucosal melanomas in humans.

Identifying the dysregulation of lncRNA expression in mucosal melanomas provides novel tools and resource that can serve as diagnostic and therapeutic targets. Here, we show by the identification of conserved dog–human lncRNAs, that they can also provide key markers in human mucosal melanomas.

## Figures and Tables

**Table 1:** List of DE lncRNAs conserved with human GENCODE non-coding genes. Genes are ordered by ascending log2-fold change (lFC).

canfam3.1+_id	Dog EnsEMBL ID	Dog Gene Biotype	lFC	Human Gene Name	Dog/Human Identity
RLOC_00034858	NA	lincRNA	−3.693	*AC016903.1*	0.770
RLOC_00028807	NA	antisense	−3.409	*AC010503.4*	0.740
RLOC_00001518	NA	lincRNA	−3.055	*EPHA1-AS1*	0.717
RLOC_00010776	NA	lincRNA	−2.974	*SOX21-AS1*	0.732
RLOC_00026330	NA	lincRNA	−2.669	*MIR29B2CHG*	0.804
RLOC_00030709	NA	antisense	−2.659	*LINC02586*	0.709
RLOC_00012258	NA	lincRNA	−2.258	*TOB1-AS1*	0.717
RLOC_00019548	NA	lincRNA	−2.214	*AL049536.1*	0.768
RLOC_00015465	NA	lincRNA	−2.201	*LINC01588*	0.794
RLOC_00011768	NA	antisense	−2.050	*AC005821.1*	0.714
RLOC_00011720	NA	antisense	−1.597	*LINC02079*	0.969
RLOC_00014809	NA	lincRNA	1.698	*AC062015.1*	0.923
RLOC_00002398	NA	antisense	1.782	*NR2F1-AS1*	0.820
RLOC_00032616	NA	lincRNA	1.868	*HOXD-AS2*	0.754
RLOC_00023326	NA	antisense	1.989	*RASSF8-AS1*	0.713
RLOC_00032620	NA	antisense	2.030	*HAGLR*	0.786
RLOC_00020381	NA	lincRNA	2.188	*TRAM2-AS1*	0.745
RLOC_00024264	NA	lincRNA	2.634	*AC133644.3*	0.979
RLOC_00021953	NA	lincRNA	3.359	*CASC15*	0.942
RLOC_00011077	NA	lincRNA	3.673	*LINC01301*	0.780
RLOC_00008433	ENSCAFG00000028700 (ZEB2-AS1)	lincRNA	3.796	*ZEB2-AS1*	0.839
RLOC_00013073	NA	lincRNA	3.797	*AC006450.3*	0.746
RLOC_00018365	NA	lincRNA	4.403	*AC090692.1*	0.755
RLOC_00022953	NA	antisense	4.910	*HOXC-AS3*	0.757
RLOC_00002254	NA	antisense	4.958	*STARD4-AS1*	0.903
RLOC_00025419	NA	lincRNA	5.758	*SNAP25-AS1*	0.987

## Supplementary Materials

The following are available online at https://www.mdpi.com/2073-4425/10/6/477/s1, Figure S1: Heatmap of xCell enrichment in 64 cell types with respect to the 52 control and tumor canine samples, Figure S2: Principal Component Analysis (PCA) of the 52 samples, Figure S3: Module-trait associations for poodle samples, Figure S4: Gene Set Enrichment Analysis, Figure S5: Breed-specific differential expression of lncRNA *COLCA1*, Table S1: Diagnostic, locations, sex, biobank_Id accessions of the 52 samples, Table S2: Summary statistics for the RNA-Seq mapping process for the 52 samples, Table S3: Characterization of the 140 DE canine lncRNAs mapped to the human genome, Table S4: Number of lncRNAs per WGCNA module, Table S5: Breed-specific DE lncRNAs. LncRNAs in poodle DE data also found in WGCNA poodle modules.
