# Supplementary material for: Genome-Wide Analysis of Long Non-Coding RNA Profiles in Canine Oral Melanomas
Source: Genes (Basel). 2019 Jun 23;10(6):477. doi: 10.3390/genes10060477 (PMC6628375; doi:10.3390/genes10060477)
Supplement: Supplementary file 1 [file genes-10-00477-s001.pdf]

Supplementary material for publication " Genome-wide analysis of long non-coding RNA profiles in canine oral melanomas" from Hitte et al.

Supplementary Figure 1. Heatmap of xcell enrichment in 64 cell type with respect to the 52 control/healthy and tumor canine samples. The scale (red/blue) colors indicates whether a sample is enriched (red) or not (blue) for a particular cell-type signature. The status (healthy in light red versus tumor in light blue) and the sampled location(s) are represented on the right panels for each sample.

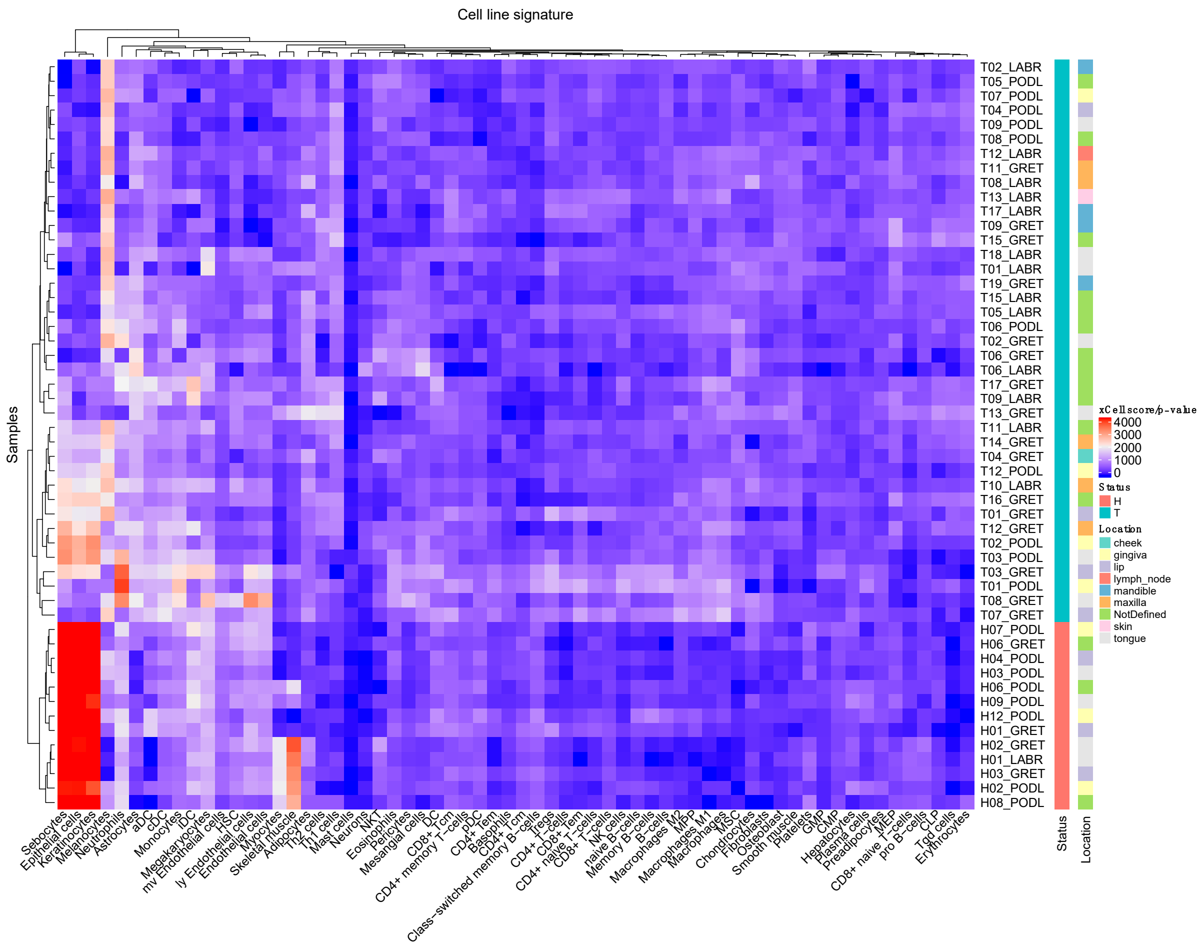

Supplementary Figure 2. Principal Component Analysis (PCA) of the 52 samples based on the **lncRNA** gene normalized counts with control/healthy sample and tumor samples in red and green, respectively.

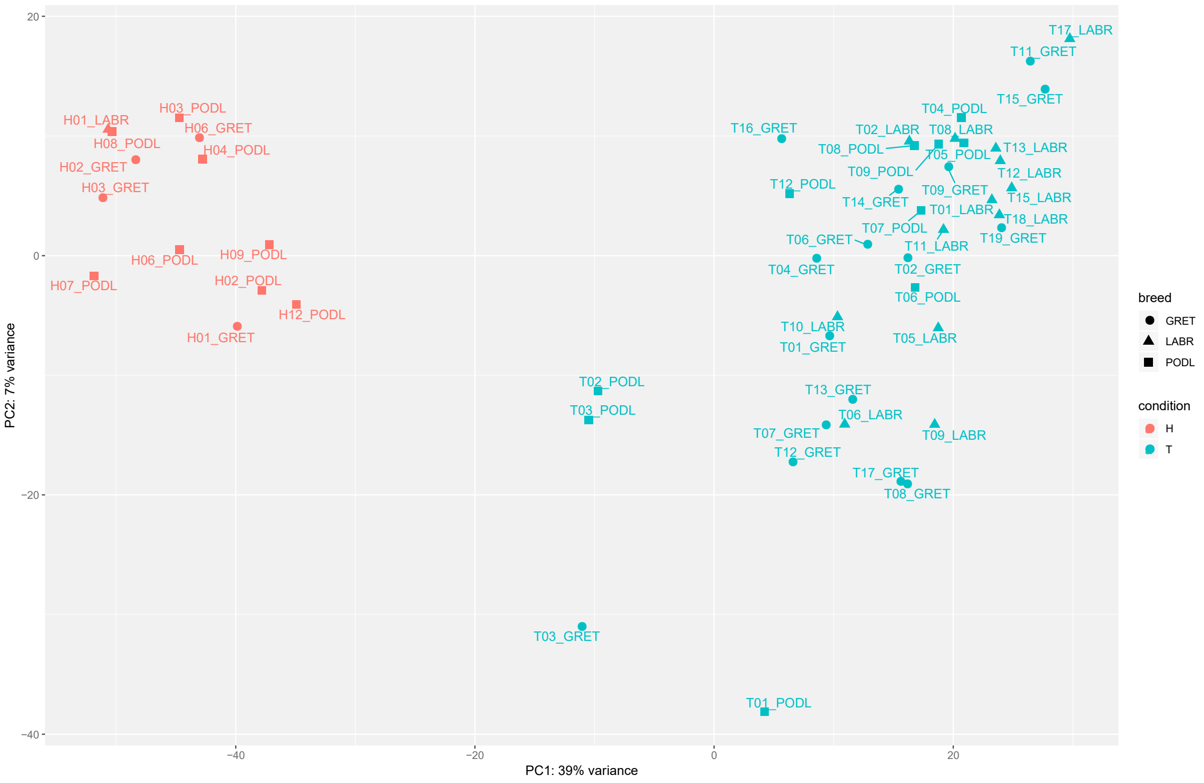

Supplementary Figure 3. Module-trait associations for poodle samples. **(a)** Each row corresponds to a ME (module eigengene), and the column to the poodle mucosal melanoma. Each cell contains the corresponding correlation and p-value with melanoma. The figure is color-coded according to the strength of the correlation (red: positive correlations; blue: negative correlations). Modules Yellow, Tan, Orange, palevioletred, darkorange and orangedred4 are positively correlated (PCC>0.8; p<6e-07). **(b)** Modules with negative correlation according to the strength of the correlation. Module Brown and Mediumorchid are the most significantly negatively correlated (p<1e-15).

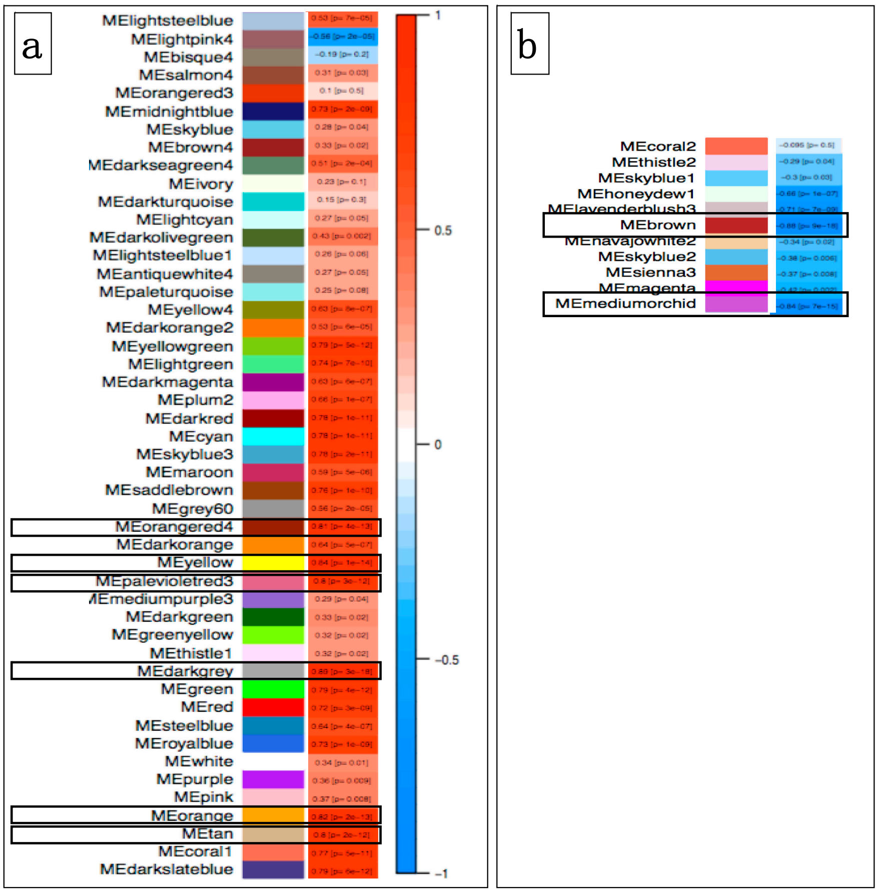

Supplementary Figure 4. GO terms (Biological Process) enriched for (a) positively correlated and (b) negatively correlated modules with oral melanoma: Top ten enriched GO items are represented.

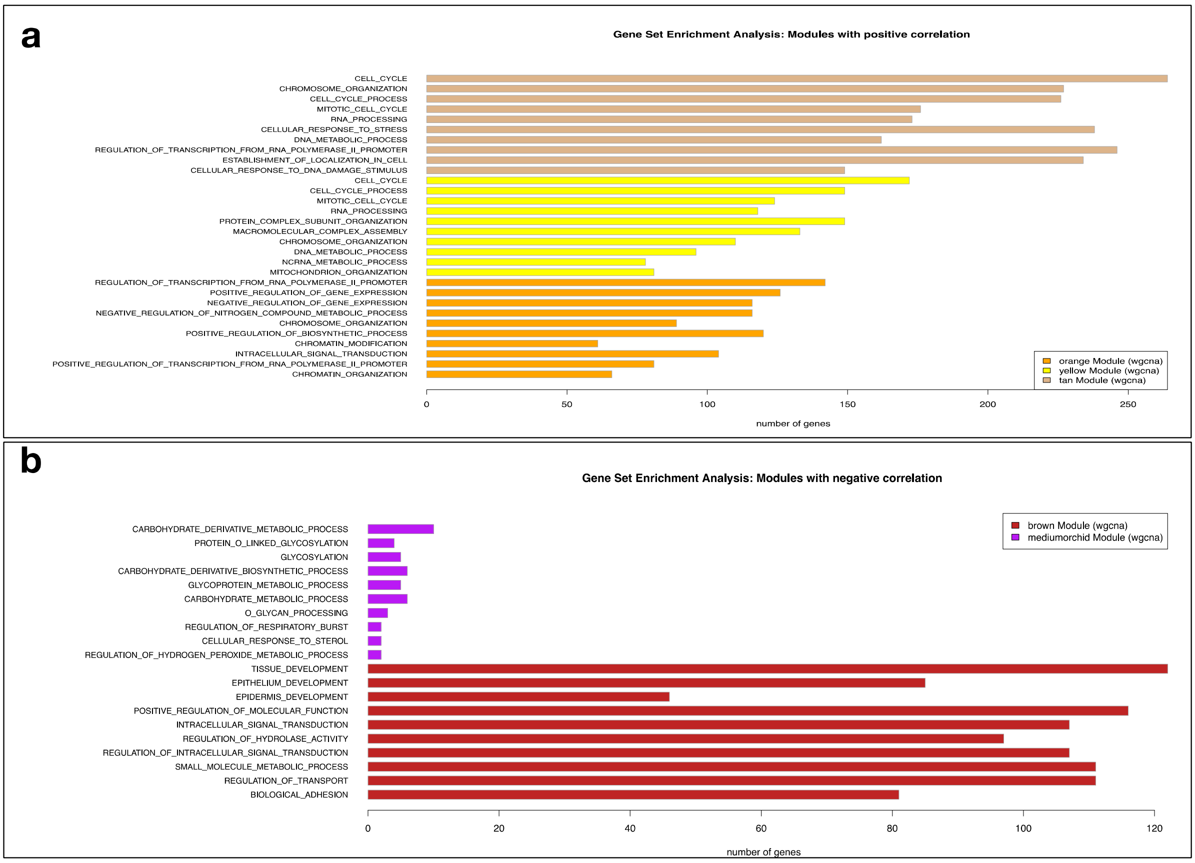

Supplementary Figure 5. Breed-specific differential expression of lncRNA RLOC_0005829 (COLCA1). Log2 of normalized count on the y-axis for control (red) versus tumor (blue) samples for golden retrievers (left panel) versus poodles (right panel)

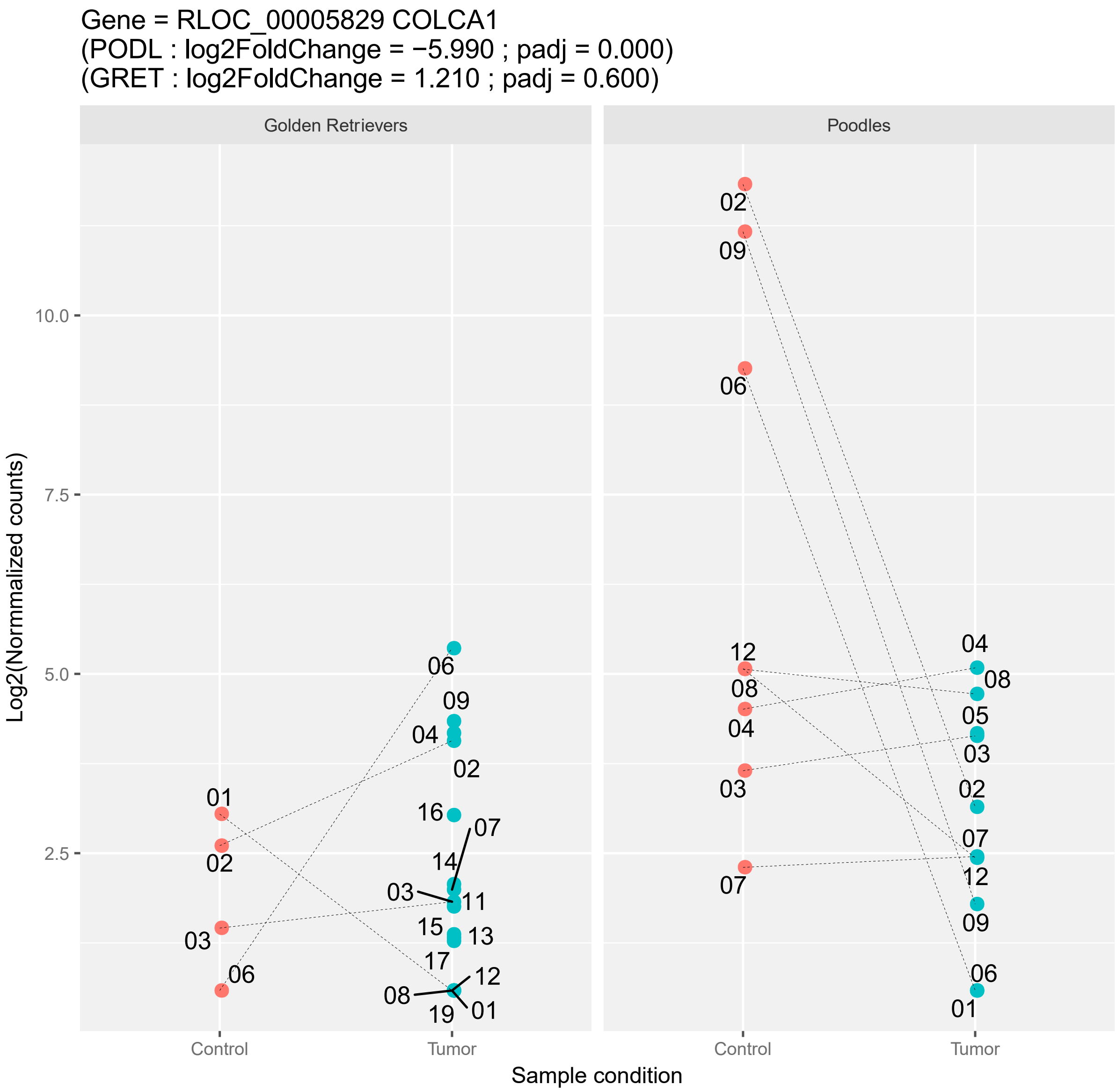

Supplementary Table 1. Diagnostic, locations, sex, biobank_Id accessions of the 52 samples:

sample_id	id_caniDNA	id_CCOGC	sex	diagnostic	primary_tumor	location
H02_PODL	3579	B00EA4K	M	oral_melanoma	NA	gingiva
T02_PODL	3579	B00EA4L	M	oral_melanoma	NA	gingiva
H03_PODL	3923	B00EA4M	F	oral_melanoma	NA	tongue
T03_PODL	3923	B00EA4N	F	oral_melanoma	NA	tongue
H04_PODL	4002	B00EA4O	NA	oral_melanoma	NA	lip
T04_PODL	4002	B00EA4P	NA	oral_melanoma	NA	lip
T01_PODL	4159	B00EA4J	M	oral_melanoma	NA	gingiva
T04_GRET	4181	B00FIO9	M	oral_melanoma	NA	cheek
T05_LABR	4207	B00FIOT	F	oral_melanoma	NA	NA
H02_GRET	4227	B00EA54	F	oral_melanoma	NA	tongue
T02_GRET	4227	B00EA55	F	oral_melanoma	NA	tongue
T02_LABR	4285	B00FIO1	M	oral_melanoma	NA	mandible
T05_PODL	4483	B00EA4R	M	oral_melanoma	NA	NA
H06_PODL	4827	B00EA4S	M	oral_melanoma	NA	NA
T06_PODL	4827	B00EA4T	M	oral_melanoma	NA	NA
H07_PODL	5224	B00EA4U	M	oral_melanoma	primary_tumor	gingiva
T07_PODL	5224	B00EA4V	M	oral_melanoma	primary_tumor	gingiva
H01_GRET	5302	B00FIOH	M	oral_melanoma	primary_tumor	lip
T01_GRET	5302	B00FIOI	M	oral_melanoma	primary_tumor	lip
H01_LABR	5720	B00EA5E	M	oral_melanoma	primary_tumor	tongue
T01_LABR	5720	B00EA5F	M	oral_melanoma	primary_tumor	tongue
H09_PODL	6528	B00EA4Y	F	oral_melanoma	primary_tumor	tongue
T09_PODL	6528	B00EA4Z	F	oral_melanoma	primary_tumor	tongue
H03_GRET	7106	B00EA56	M	oral_melanoma	NA	lip
T03_GRET	7106	B00EA57	M	oral_melanoma	NA	lip
H12_PODL	7855	B00EA52	F	oral_melanoma	NA	gingiva
T12_PODL	7855	B00EA53	F	oral_melanoma	NA	gingiva
H06_GRET	10271	B00EA7P	F	oral_melanoma	NA	NA
T06_GRET	10271	B00EA7O	F	oral_melanoma	NA	NA
T06_LABR	10501	B00EA7Q	M	oral_melanoma	NA	NA
T07_GRET	CCOGC	CCB010032	NA	oral_melanoma	primary_tumor	lip
T08_GRET	CCOGC	CCB010284	NA	oral_melanoma	primary_tumor	tongue
T08_LABR	CCOGC	CCB010318	NA	oral_melanoma	primary_tumor	maxilla
T09_GRET	CCOGC	CCB010326	NA	oral_melanoma	primary_tumor	mandible
T09_LABR	CCOGC	CCB030038	NA	melanoma	NA	NA
T10_LABR	CCOGC	CCB030039	NA	oral_melanoma	primary_tumor	maxilla
T11_GRET	CCOGC	CCB030075	NA	oral_melanoma	primary_tumor	maxilla
T11_LABR	CCOGC	CCB030097	NA	melanoma	NA	NA
T12_GRET	CCOGC	CCB030120	NA	oral_melanoma	primary_tumor	maxilla
T12_LABR	CCOGC	CCB030183	NA	oral_melanoma	primary_tumor	lymph_node

T13_GRET	CCOGC	CCB030165	NA	oral_melanoma	primary_tumor	tongue
T13_LABR	CCOGC	CCB030269	NA	oral_melanoma	primary_tumor	skin
T14_GRET	CCOGC	CCB030201	NA	oral_melanoma	primary_tumor	maxilla
T15_GRET	CCOGC	CCB030227	NA	oral_melanoma	primary_tumor	NA
T15_LABR	CCOGC	CCB050023	NA	melanoma	NA	NA
T16_GRET	CCOGC	CCB030342	NA	melanoma	NA	NA
T17_GRET	CCOGC	CCB050061	NA	melanoma	NA	NA
T17_LABR	CCOGC	CCB050096	NA	oral_melanoma	primary_tumor	mandible
T18_LABR	CCOGC	CCB050228	NA	oral_melanoma	primary_tumor	tongue
T19_GRET	CCOGC	CCB030273	NA	oral_melanoma	primary_tumor	mandible
H08_PODL	5223	B00EA4W	NA	melanoma	NA	NA
T08_PODL	5223	B00EA4X	NA	melanoma	NA	NA

Supplementary Table 2. Summary statistics the RNASeq mapping processe for the 52 samples:

sample_id	total_reads	mapped_reads	pct_mapped
H01_GRET	114905418	109642636	95.4199
H01_LABR	102060738	97938964	95.9614
H02_GRET	116303444	111676186	96.0214
H02_PODL	115579956	108119950	93.5456
H03_GRET	101617962	95662328	94.1392
H03_PODL	95705334	90740286	94.8122
H04_PODL	100513348	95351416	94.8644
H06_GRET	123108392	117705856	95.6116
H06_PODL	122237464	116835596	95.5808
H07_PODL	104765400	100429218	95.8611
H08_PODL	127577638	122485416	96.0085
H09_PODL	122256708	115618482	94.5703
H12_PODL	152311560	142131396	93.3162
T01_GRET	119742150	113552482	94.8308
T01_LABR	103997760	98375058	94.5934
T01_PODL	125795232	115560294	91.8638
T02_GRET	111188146	104754146	94.2134
T02_LABR	117748272	112530866	95.569
T02_PODL	94925824	88850866	93.6003
T03_GRET	130238842	116883454	89.7455
T03_PODL	86720270	82059442	94.6254
T04_GRET	102774558	98010058	95.3641
T04_PODL	132289310	125515066	94.8792
T05_LABR	113531352	107357078	94.5616
T05_PODL	96974182	91707760	94.5693
T06_GRET	106128548	100660864	94.8481
T06_LABR	97256470	92496866	95.1061
T06_PODL	101797096	95964134	94.27
T07_GRET	105498510	98986656	93.8275
T07_PODL	94957170	89827360	94.5978
T08_GRET	106548606	100440800	94.2676
T08_LABR	102694776	96991868	94.4467
T08_PODL	100125334	95125576	95.0065
T09_GRET	120683698	113721778	94.2313
T09_LABR	110032172	104250598	94.7456
T09_PODL	99976306	93809504	93.8317
T10_LABR	108000438	102370000	94.7867
T11_GRET	121441158	115316112	94.9564
T11_LABR	108637932	103477814	95.2502
T12_GRET	122557258	116710318	95.2292
T12_LABR	114603244	108376544	94.5667

T12_PODL	95565878	89492478	93.6448
T13_GRET	127578536	121658572	95.3597
T13_LABR	87928130	83859136	95.3724
T14_GRET	112779000	106870816	94.7613
T15_GRET	110387888	105013088	95.131
T15_LABR	117853920	112352020	95.3316
T16_GRET	92061920	86920024	94.4147
T17_GRET	106246074	100475354	94.5685
T17_LABR	86790284	81724812	94.1635
T18_LABR	105127638	98654102	93.8422
T19_GRET	100523080	95593834	95.0964

Supplementary Table 3 Characterization of the 140 DE canine lncRNA mapped to the human genome:

canfam3.1+_id	dog_ensembl_id	dog_gene_name	dog_biotype	baseMean	log2FoldChange	padj	human_gencode_name	human_minimap_grch38_location	blastId
RLOC_00001826	NA	NA	antisense_mRNA	3.517	-6.6	0.001	NA	8:19400195-19400456	0.792
RLOC_00020145	NA	NA	lincRNA	4.214	-5.454	0.003	NA	6:37127933-37129124	0.713
RLOC_00013063	NA	NA	antisense_mRNA	7.741	-5.31	0.001	NA	9:124482877-124483033	0.91
RLOC_00011253	NA	NA	antisense_mRNA	2.327	-5.309	0.003	NA	8:78661484-78661610	0.898
RLOC_00009916	NA	NA	lincRNA	22.705	-5.272	0	NA	1:10806922-10808221	0.995
RLOC_00003953	NA	NA	lincRNA	139.13	-5.017	0	NA	6:167699625-167699795	0.876
RLOC_00036091	NA	NA	lincRNA	26.722	-4.733	0	NA	3:194061717-194061912	0.908
RLOC_00008917	NA	NA	antisense_mRNA	85.695	-4.619	0	NA	5:139418765-139439872	0.747
RLOC_00011395	NA	NA	lincRNA	47.425	-4.268	0	NA	8:94613434-94613666	0.991
RLOC_00010651	NA	NA	lincRNA	70.931	-4.178	0.016	NA	13:73573243-73631916	0.987
RLOC_00023851	NA	NA	lincRNA	75.928	-4.133	0	NA	1:226262092-226263452	0.713
RLOC_00002766	NA	NA	lincRNA	9.026	-4.04	0.004	NA	15:85828889-85829657	0.764
RLOC_00032101	NA	NA	antisense_mRNA	33.778	-4.007	0	NA	3:184746767-184748790	0.755
RLOC_00024752	NA	NA	lincRNA	507.008	-3.985	0.001	NA	1:152946212-152950093	0.722
RLOC_00016405	NA	NA	lincRNA	13.174	-3.974	0.013	NA	15:38699513-38704947	0.746

RLOC_00009407	NA	NA	lincRNA	20.422	-3.918	0.001	NA	16:49861297-49862988	0.726
RLOC_00036225	NA	NA	lincRNA	5.583	-3.703	0.004	NA	3:29279923-29281756	0.814
RLOC_00034858	NA	NA	lincRNA	26.118	-3.693	0.001	AC016903.1	2:204467218-204476382	0.77
RLOC_00003240	NA	NA	lincRNA	7.407	-3.655	0.004	NA	4:26021479-26024276	0.733
RLOC_00031699	NA	NA	antisense_mRNA	2.357	-3.629	0.014	NA	7:33533142-33535108	0.774
RLOC_00000822	NA	NA	antisense_mRNA	112.529	-3.52	0	NA	X:101349026-101371719	0.78
RLOC_00003439	NA	NA	lincRNA	207.919	-3.517	0	NA	18:70469817-70475663	0.986
RLOC_00028807	NA	NA	antisense_mRNA	353.137	-3.409	0	AC010503.4	19:6460555-6470378	0.74
RLOC_00027293	NA	NA	antisense_mRNA	33.738	-3.404	0.005	NA	18:26389353-26390048	0.781
RLOC_00021053	NA	NA	lincRNA	17.176	-3.346	0	NA	8:116321704-116387023	0.711
RLOC_00020119	NA	NA	antisense_mRNA	11.089	-3.327	0.007	NA	6:36138249-36140394	0.725
RLOC_00023406	NA	NA	antisense_mRNA	3.847	-3.304	0.012	NA	12:18945625-19066744	0.709
RLOC_00017738	NA	NA	antisense_mRNA	10.453	-3.255	0.002	NA	9:97773665-97774215	0.811
RLOC_00007749	NA	NA	lincRNA	4.853	-3.069	0.021	NA	12:102502194-102633029	0.724
RLOC_00012058	NA	NA	antisense_mRNA	5.652	-3.057	0.017	NA	17:40980564-40985072	0.789
RLOC_00001518	NA	NA	lincRNA	78.776	-3.055	0.011	EPHA1-AS1	7:143413268-143416558	0.717
RLOC_00010776	NA	NA	lincRNA	9.665	-2.974	0.003	SOX21-AS1	13:94713461-94718179	0.732
RLOC_0003086	NA	NA	lincRNA	11.6	-2.927	0.002	NA	2:177318672-177345751	0.731

4				53					
RLOC_00010214	NA	NA	lincRNA	908.67	-2.849	0.008	NA	21:37546740-37547661	0.99
RLOC_00022985	NA	NA	lincRNA	9.462	-2.783	0.02	NA	12:53242825-53245684	0.781
RLOC_00005492	NA	NA	lincRNA	51.276	-2.728	0.012	NA	11:129311990-129312596	0.708
RLOC_00023961	NA	NA	antisense_mRNA	13.669	-2.685	0	NA	20:59098490-59098862	0.794
RLOC_00026330	NA	NA	lincRNA	11.054	-2.669	0.022	MIR29B2CHG	1:207821308-207824899	0.804
RLOC_00030709	NA	NA	antisense_mRNA	99.031	-2.659	0.001	LINC02586	1:110207838-110213072	0.709
RLOC_00004345	NA	NA	antisense_mRNA	23.181	-2.596	0.004	NA	9:68533592-68537962	0.712
RLOC_00002364	NA	NA	lincRNA	4.344	-2.57	0.009	NA	5:96150143-96159185	0.794
RLOC_00007180	NA	NA	antisense_mRNA	4.719	-2.459	0.014	NA	1:36321280-36322814	0.755
RLOC_00011453	NA	NA	antisense_mRNA	37.809	-2.452	0.012	NA	17:82036855-82037018	0.933
RLOC_00004293	NA	NA	lincRNA	12.243	-2.382	0.026	NA	9:72474981-72482475	0.938
RLOC_00025023	NA	NA	antisense_mRNA	74.549	-2.365	0.002	NA	3:113289143-113298453	0.708
RLOC_00019902	NA	NA	lincRNA	8.806	-2.341	0.009	NA	10:88866630-88866996	0.785
RLOC_00021785	NA	NA	lincRNA	10.668	-2.333	0.018	NA	6:6875783-6882303	0.73
RLOC_00003692	NA	NA	antisense_mRNA	24.557	-2.317	0	NA	19:5576518-5576945	0.998
RLOC_00013173	NA	NA	lincRNA	4.473	-2.279	0.036	NA	12:56773889-56774167	0.792
RLOC_00032064	NA	NA	antisense_mRNA	122.45	-2.267	0.003	NA	17:42797643-42798699	0.996
RLOC_00012258	NA	NA	lincRNA	5.215	-2.258	0.032	TOB1-AS1	17:50909366-50910825	0.717
RLOC_0	NA	NA	lincRNA	89	-2.254	0.01	NA	14:72969456-73024912	0.851

0015461				.328					
RLOC_00018322	NA	NA	lincRNA	21.454	-2.254	0.012	NA	11:44055902-44056406	0.73
RLOC_00019548	NA	NA	lincRNA	14.417	-2.214	0.035	AL049536.1	22:27413684-27436165	0.768
RLOC_00015465	NA	NA	lincRNA	21.455	-2.201	0.001	LINC01588	14:50037486-50039627	0.794
RLOC_00018883	NA	NA	antisense_mRNA	1.375	-2.195	0.047	NA	11:66720065-66722150	0.707
RLOC_00011750	NA	NA	antisense_mRNA	94.508	-2.179	0	NA	17:46304000-46306307	0.726
RLOC_00005489	NA	NA	lincRNA	25.88	-2.17	0.035	NA	11:129583155-129699041	0.947
RLOC_00026547	NA	NA	lincRNA	36.763	-2.159	0	NA	1:179019960-179024746	0.975
RLOC_00010989	NA	NA	antisense_mRNA	3.811	-2.12	0.046	NA	8:48763828-48764820	0.782
RLOC_00016055	NA	NA	antisense_mRNA	237.217	-2.107	0.002	NA	14:99510739-99512729	0.861
RLOC_00029149	NA	NA	lincRNA	14.387	-2.052	0.013	NA	10:91574959-91575491	0.757
RLOC_00011768	NA	NA	antisense_mRNA	17.797	-2.05	0.017	AC005821.1	17:62705998-62725162	0.714
RLOC_00019077	NA	NA	antisense_mRNA	50.134	-2.022	0.017	NA	11:62355541-62358073	0.994
RLOC_00003758	NA	NA	lincRNA	55.632	-2.009	0.039	NA	6:148193491-148197294	0.743
RLOC_00010227	NA	NA	lincRNA	64.73	-1.999	0.011	NA	21:38744031-38745414	0.812
RLOC_00021073	NA	NA	lincRNA	20.272	-1.972	0.038	NA	8:119281368-119281906	0.821
RLOC_00035571	NA	NA	antisense_mRNA	27.935	-1.965	0.002	NA	3:141738158-141738558	0.88
RLOC_00023318	NA	NA	lincRNA	20.213	-1.961	0.002	NA	12:26833827-26835177	0.737
RLOC_0000856	NA	NA	antisense_mRNA	21.6	-1.942	0	NA	X:136874171-136874391	1

0				51					
RLOC_00020746	NA	NA	antisense_mRNA	14.802	-1.927	0.034	NA	6:105256664-105258650	0.785
RLOC_00033106	NA	NA	lincRNA	171.795	-1.894	0.004	NA	10:75238910-75239662	0.985
RLOC_00027931	NA	NA	lincRNA	18.323	-1.876	0.028	NA	3:2094847-2098421	0.744
RLOC_00011886	NA	NA	antisense_mRNA	165.893	-1.859	0.005	NA	17:32090743-32091946	0.994
RLOC_00029252	NA	NA	antisense_mRNA	43.737	-1.836	0.009	NA	10:97707984-97714141	0.945
RLOC_00015636	NA	NA	antisense_mRNA	23.198	-1.821	0.014	NA	17:51159977-51161996	0.998
RLOC_00007862	NA	NA	lincRNA	7.677	-1.816	0.043	NA	16:30067332-30070413	0.976
RLOC_00035575	NA	NA	antisense_mRNA	66.945	-1.813	0.012	NA	3:141873105-141874753	0.745
RLOC_00009024	NA	NA	lincRNA	120.389	-1.778	0.023	NA	5:146085493-146092534	0.938
RLOC_00034833	NA	NA	antisense_mRNA	54.424	-1.697	0.008	NA	2:202632868-202635507	0.763
RLOC_00000565	NA	NA	lincRNA	1841.412	-1.689	0	NA	X:56728711-56817942	0.989
RLOC_00020569	NA	NA	antisense_mRNA	40.078	-1.683	0.003	NA	11:2486220-2630113	0.722
RLOC_00031417	NA	NA	antisense_mRNA	17.793	-1.674	0.001	NA	7:94407813-94508796	0.718
RLOC_00015328	NA	NA	lincRNA	29.81	-1.659	0.019	NA	14:34060843-34064454	0.735
RLOC_00011722	NA	NA	antisense_mRNA	70.983	-1.642	0.046	NA	9:4711865-4741093	0.995
RLOC_00033235	NA	NA	antisense_mRNA	197.614	-1.602	0.001	NA	5:177402625-177519663	0.998
RLOC_0001172	NA	NA	antisense_mRNA	233.	-1.597	0.018	LINC02079	17:39026607-39026941	0.969

0				011					
RLOC_00011759	NA	NA	lincRNA	40.391	-1.58	0.041	NA	17:62303605-62304895	0.836
RLOC_00035703	NA	NA	lincRNA	487.729	-1.571	0	NA	3:153152240-153159756	0.729
RLOC_00022658	NA	NA	antisense_mRNA	254.48	1.561	0	NA	11:8947662-8950777	0.808
RLOC_00014809	NA	NA	lincRNA	4.21	1.698	0.041	AC062015.1	2:226184949-226186263	0.923
RLOC_00023934	ENSCAFG00000003742	NA	antisense_mRNA	17.149	1.736	0	NA	9:33026484-33038903	0.924
RLOC_00002398	NA	NA	antisense_mRNA	86.464	1.782	0.003	NR2F1-AS1	5:93409596-93584109	0.82
RLOC_00029966	NA	NA	lincRNA	20.164	1.801	0.037	NA	7:6273510-6277810	0.744
RLOC_00032616	NA	NA	lincRNA	67.106	1.868	0.014	HOXD-AS2	2:176134485-176136721	0.754
RLOC_00023326	NA	NA	antisense_mRNA	3.806	1.989	0.029	RASSF8-AS1	12:25833963-25958356	0.713
RLOC_00015703	NA	NA	lincRNA	472.906	1.992	0.023	NA	14:68766615-68768450	0.704
RLOC_00011700	NA	NA	antisense_mRNA	74.338	2.022	0	NA	17:73168411-73189776	0.81
RLOC_00032620	NA	NA	antisense_mRNA	159.885	2.03	0.023	HAGLR	2:176175750-176177638	0.786
RLOC_00034734	NA	NA	antisense_mRNA	408.313	2.084	0.003	NA	2:196716175-196728386	0.872
RLOC_00002175	NA	NA	lincRNA	46.34	2.112	0.004	NA	8:12393036-12393416	0.999
RLOC_00020381	NA	NA	lincRNA	86.589	2.188	0	TRAM2-AS1	6:52579196-52599155	0.745
RLOC_00002920	NA	NA	lincRNA	63.512	2.227	0.016	NA	16:69339826-69342312	0.907
RLOC_0003241	NA	NA	lincRNA	9.23	2.31	0.02	NA	2:156803533-156806507	0.729

2									
RLOC_00008584	NA	NA	lincRNA	3.453	2.378	0.032	NA	5:123408497-123411660	0.987
RLOC_00026713	NA	NA	antisense_mRNA	6.2	2.41	0.046	NA	1:166935115-166937358	0.798
RLOC_00033708	NA	NA	lincRNA	2.1	2.467	0.037	NA	5:37841298-37842236	0.854
RLOC_00021152	NA	NA	lincRNA	21.889	2.482	0.013	NA	8:125946795-125947565	0.915
RLOC_00004130	NA	NA	antisense_mRNA	24.493	2.561	0	NA	9:96417624-96417828	0.814
RLOC_00024964	NA	NA	lincRNA	10.358	2.627	0.04	NA	3:108036154-108037304	0.765
RLOC_00024264	NA	NA	lincRNA	936.056	2.634	0	AC133644.3	2:87455277-87622920	0.979
RLOC_00034277	NA	NA	antisense_mRNA	21.833	2.64	0.013	NA	1:151982914-151986253	0.85
RLOC_00016416	ENSCAFG00000032478	NA	antisense_mRNA	2.714	2.674	0.044	NA	18:70325820-70327011	0.801
RLOC_00004029	NA	NA	lincRNA	63.999	2.963	0.014	NA	6:121868301-121903730	0.992
RLOC_00034166	NA	NA	antisense_mRNA	15.563	2.966	0.001	NA	4:107913565-107917791	0.994
RLOC_00028392	NA	NA	lincRNA	30.578	3	0.001	NA	19:18800557-18801451	0.706
RLOC_00015479	NA	NA	lincRNA	43.107	3.123	0.001	NA	14:50839525-50858164	0.799
RLOC_00015048	NA	NA	antisense_mRNA	3.613	3.21	0.025	NA	2:240591845-240592157	0.821
RLOC_00014117	NA	NA	lincRNA	29.158	3.239	0.003	NA	2:58518658-58547029	0.737
RLOC_00029138	NA	NA	lincRNA	9.138	3.239	0.015	NA	10:90683230-90684489	0.71
RLOC_00031589	NA	NA	lincRNA	3.396	3.24	0.008	NA	7:25387592-25397227	0.781
RLOC_00021953	NA	NA	lincRNA	73.58	3.359	0	CASC15	6:21666398-21885801	0.942

RLOC_00012494	NA	NA	lincRNA	16.122	3.553	0.004	NA	17:33294457-33316319	0.875
RLOC_00026556	NA	NA	lincRNA	7.631	3.663	0.003	NA	6:117999273-117999586	0.756
RLOC_00011077	NA	NA	lincRNA	2.595	3.673	0.023	LINC01301	8:60403489-60405252	0.78
RLOC_00008433	ENSCAFG00000028700,ENSCAFG00000029122	ZEB2_AS1_4,ZEB2_AS1_3	lincRNA	21.793	3.796	0	ZEB2-AS1	2:144520485-144522161	0.839
RLOC_00013073	NA	NA	lincRNA	18.168	3.797	0.015	AC006450.3	9:124007555-124011599	0.746
RLOC_00035720	NA	NA	lincRNA	63.572	3.87	0.003	NA	6:103205226-103205781	1
RLOC_00024794	ENSCAFG00000030382	NA	lincRNA	113.264	3.936	0	NA	1:111726510-111727221	0.713
RLOC_00016582	NA	NA	antisense_mRNA	68.65	4.185	0	NA	15:48296762-48299154	0.753
RLOC_00009057	NA	NA	antisense_mRNA	4.25	4.289	0.001	NA	5:55218929-55223040	0.752
RLOC_00034218	NA	NA	antisense_mRNA	4.48	4.36	0.008	NA	4:112508644-112519940	0.743
RLOC_00018365	NA	NA	lincRNA	253.057	4.403	0	AC090692.1	11:35912935-35915548	0.755
RLOC_00001229	NA	NA	antisense_mRNA	12.982	4.513	0.005	NA	X:140503528-140506836	0.75
RLOC_00008492	NA	NA	antisense_mRNA	63.181	4.861	0	NA	2:151671008-151675686	0.75
RLOC_00005892	NA	NA	antisense_mRNA	5.396	4.883	0.001	NA	2:9584266-9630510	0.796
RLOC_00022953	NA	NA	antisense_mRNA	6.571	4.91	0.01	HOXC-AS3	12:53981706-53984833	0.757
RLOC_00002254	NA	NA	antisense_mRNA	22.293	4.958	0.007	STARD4-AS1	5:111730123-111731168	0.903
RLOC_00025419	NA	NA	lincRNA	6.199	5.758	0.041	SNAP25-AS1	20:10060634-10219509	0.987

RLOC_0 001673 2	NA	NA	lincRNA	78 .1 5	5.923	0	NA	15:60843611-60847670	0.735

Supplementary Table 4. Number of lncRNAs per WGCNA modules.

lncRNA	Module_color_assignment
24	antiquewhite4
10	bisque4
529	brown
50	brown4
22	coral1
22	coral2
154	cyan
124	darkgreen
136	darkgrey
100	darkmagenta
29	darkolivegreen
134	darkorange
37	darkorange2
147	darkred
85	darkseagreen4
66	darkslateblue
234	darkturquoise
337	green
219	greenyellow
162	grey60
268	honeydew1
40	ivory
47	lavenderblush3
172	lightcyan
153	lightgreen
39	lightpink4
17	lightsteelblue
48	lightsteelblue1
297	magenta
27	maroon
18	mediumorchid
85	mediumpurple3
222	midnightblue
43	navajowhite2
627	orange
16	orangered3
77	orangered4
93	paleturquoise
45	palevioletred3
147	pink
57	plum2
318	purple

287	red
180	royalblue
73	saddlebrown
34	salmon4
75	sienna3
87	skyblue
15	skyblue1
19	skyblue2
32	skyblue3
41	steelblue
408	tan
28	thistle1
65	thistle2
152	white
97	yellow
25	yellow4
71	yellowgreen

Supplementary Table 5. Breed-specific DE lncRNAs. LncRNAs in poodles (PODL) are also found in WGCNA poodle modules.

Id	Biotype	Normalized mean counts	lFC	p.adj	breed
RLOC_00026622	antisense_mRNA	22.311	-3.392	0.000	PODL
RLOC_00030862	lincRNA	10.636	-2.800	0.004	PODL
RLOC_00027767	lincRNA	8.954	1.502	0.008	PODL
RLOC_00023881	antisense_mRNA	94.765	1.567	0.010	PODL
RLOC_00020800	antisense_mRNA	33.681	1.835	0.027	PODL
RLOC_00012324	lincRNA	12.754	1.904	0.005	PODL
RLOC_00012791	antisense_mRNA	23.892	1.922	0.001	PODL
RLOC_00017181	antisense_mRNA	5.893	1.947	0.033	PODL
RLOC_00007411	antisense_mRNA	13.398	1.959	0.002	PODL
RLOC_00021127	antisense_mRNA	39.942	1.960	0.003	PODL
RLOC_00034770	antisense_mRNA	66.448	2.570	0.000	PODL
RLOC_00009497	lincRNA	6.248	-3.356	0.030	GRET
RLOC_00014840	antisense_mRNA	5.784	-3.263	0.019	GRET
RLOC_00028757	antisense_mRNA	5.839	-2.365	0.007	GRET
RLOC_00007259	lincRNA	123.353	2.502	0.001	GRET
RLOC_00036011	lincRNA	5.198	3.100	0.037	GRET
RLOC_00026641	lincRNA	5.099	4.672	0.021	GRET
RLOC_00019703	lincRNA	6.052	5.082	0.045	GRET
